# The rectification control and physiological relevance of potassium channel OsAKT2

**DOI:** 10.1093/plphys/kiab462

**Published:** 2021-10-02

**Authors:** Ya-Nan Huang, Shun-Ying Yang, Jun-Lin Li, Shao-Fei Wang, Jia-Jin Wang, Dong-Li Hao, Yan-Hua Su

**Affiliations:** 1 State Key Laboratory of Soil and Sustainable Agriculture, Institute of Soil Science, Chinese Academy of Sciences, Nanjing 210008, China; 2 University of Chinese Academy of Sciences, Beijing 100049, China; 3 Shandong Institute of Sericulture, Yantai 264002, China

## Abstract

AKT2 potassium (K^+^) channels are members of the plant Shaker family which mediate dual-directional K^+^ transport with weak voltage-dependency. Here we show that OsAKT2 of rice (*Oryza sativa*) functions mainly as an inward rectifier with strong voltage-dependency and acutely suppressed outward activity. This is attributed to the presence of a unique K191 residue in the S4 domain. The typical bi-directional leak-like property was restored by a single K191R mutation, indicating that this functional distinction is an intrinsic characteristic of OsAKT2. Furthermore, the opposite R195K mutation of AtAKT2 changed the channel to an inward-rectifier similar to OsAKT2. OsAKT2 was modulated by OsCBL1/OsCIPK23, evoking the outward activity and diminishing the inward current. The physiological relevance in relation to the rectification diversity of OsAKT2 was addressed by functional assembly in the Arabidopsis (*Arabidopsis thaliana*) *akt2* mutant. Overexpression (OE) of *OsAKT2* complemented the K^+^ deficiency in the phloem sap and leaves of the mutant plants but did not significantly contribute to the transport of sugars. However, the expression of *OsAKT2-K191R* overcame both the shortage of phloem K^+^ and sucrose of the *akt2* mutant, which was comparable to the effects of the OE of *AtAKT2*, while the expression of the inward mutation *AtAKT2-R195K* resembled the effects of *OsAKT2*. Additionally, OE of *OsAKT2* ameliorated the salt tolerance of Arabidopsis.

## Teaser

The presence of a unique K191 residue retains the activity of rice potassium channel OsAKT2 mainly as an inward rectifier (Mode I) that emphasizes its in planta role of phloem K^+^ translocation.

## Introduction

AKT2 channels belong to a particular K_weak_ group of plant Shaker potassium (K^+^) channels and typically mediate both K^+^ uptake and efflux in a weakly voltage-dependent manner (Philippar et al., 1999; Lacombe et al., 2000; Ache et al., 2001; Shen et al., 2020). In this sense, AKT2 is also referred to as a “leak-like” K^+^ channel (Dreyer et al., 2001; Ragel et al., 2019). Despite the functional diversity among the plant Shaker channels of the *K*_in_ (KAT- and AKT-type), *K*_out_ (SKOR and GORK), or *K*_weak_ (AKT2-type) subgroups, they share the fundamental structural similarities, including six transmembrane segments (S1–S6), a signature pore motif located between S5 and S6, a putative cyclic nucleotide-binding domain, and a cytosolic C-terminus of varied length (Véry and Sentenac, 2003; Nieves-Cordones et al., 2014; Véry et al., 2014). Except for the voltage-gated *K*_in_ channels of the KAT-type, an additional ankyrin (ANK) repeat domain is present to the C-terminus of the rest of plant Shakers, including AKT2 (Véry and Sentenac, 2003).

Though not strictly voltage-dependent and somehow leak like, AKT2 behaves as a K^+^ selective channel as evidenced by the predominant permeability to K^+^ over other monovalent ions, and the channel activity is specifically blocked by K^+^ channel blockers Ba^2+^, Cs^+^, or tetraethylammonium (Marten et al., 1999; Lacombe et al., 2000). Therefore, the mode-of-action of the dual-rectification associated with AKT2 has attracted considerable interest over the past years. It has been suggested that AtAKT2 changes its gating mode/rectification pending on the accumulation of expression levels in COS and CHO cells (Dreyer et al., 2001) or engages a switch between two different gating modes via a reversible phosphorylation mechanism (Michard et al., 2005a; Sandmann et al., 2011). In the resting gating modes, nonphosphorylated (gating mode 1) AtAKT2 mediates time- and voltage-dependent inward-rectifying K^+^ currents and lacks its outward component. Upon protein kinase A-mediated (PKA-mediated) phosphorylation, AtAKT2 switches to gating Mode 2 characterized by the presence of nonrectifying leak-like K^+^ currents and allows K^+^ efflux (Michard et al., 2005a, 2005b). Two putative PKA phosphorylation sites (S210 and S329) are suggested to be involved in the switching of gating modes (Michard et al., 2005a). Additionally, the substitution of the K197 in the S4 segment of AtAKT2 by a serine or aspartate abolishes the “open-lock” characteristic and converts AtAKT2 into an inward-rectifying channel like AtKAT1 (Michard et al., 2005b). These three residues are conserved among *K*_weak_ channels and correspond, respectively, to K193, N206, and S326 of the rice OsAKT2 (Shen et al., 2020).

The voltage-insensitive K^+^ efflux activity AtAKT2 has been suggested to contain a physiological relevance in Arabidopsis: the efflux of K^+^ prevents continuing depolarization of the phloem cell which is caused by the influx of H^+^, and could then drive sucrose uptake via sucrose/H^+^ symporters (Gajdanowicz et al., 2011). Thus, an important role of AKT2 is to assist the downward transport of photosynthetic sugars by electro-energizing the plasma membrane of phloem cells for efficient loading of sugars (Dreyer et al., 2017). In Arabidopsis, knockout of the AtAKT2 function leads to reduced growth and retarded development of the *akt2* mutant plants (Dennison et al., 2001; Gajdanowicz et al., 2011). Although not statistically significant, the K^+^ concentration in the phloem sap of *akt2* mutant plants shows a decreasing tendency (Deeken et al., 2002). A more straightforward observation is the reduced K^+^ dependence of the phloem membrane potential of the *akt2* mutant that subsequently leads to halfway reduced sucrose content in the phloem (Deeken et al., 2002). Therefore, in Arabidopsis, the role of AtAKT2 is linked to the phloem transport of sugars that affects plant growth and development.

The findings that CBL1 (or CBL9)/CIPK23 activates AtAKT1 and enhances its K^+^ uptake activity (Xu et al., 2006) presents a clear molecular pathway, through which the activity of K^+^ channels is modulated. More recently, by X-ray crystallography, Sanchez-Barrena et al. (2020) resolved the physical interaction between CIPK23 and the ANK domain in the cytosolic side of AtAKT1 that is essential for the activation of AtAKT1 by the CBL/CIPK complexes. Therefore, the presence of the ANK repeat domains is a prerequisite for the CBL/CIPK modulation, and the diversity of the ANK domains determines the spectrum or specificity of the interacting CBL/CIPK species (Sánchez-Barrena et al., 2020; Thiruppathi, 2020). For instance, the activity of AtAKT2 is enhanced in oocytes by the co-expression of the AtCBL4/CIPK6 module (Held et al., 2011); the AtCBL1/CIPK5 complex is responsible for AtGORK activation in response to wounding-induced stomatal closure (Förster et al., 2019); and in rice roots, OsAKT1 is promoted by the OsCBL1/OsCIPK23 complex (Li et al., 2014). In addition to K^+^ channels, the Arabidopsis nitrate transporter AtNRT1.1 (Ho et al., 2009; Wang et al., 2020), ammonium transporter AtAMT1 (Straub et al., 2017), and high-affinity K^+^ uptake transporter AtHAK5 (Ragel et al., 2015) can also be targets of the CBL1 (or CBL9)/CIPK23 module. Thus the CBL1/CIPK23 module plays a broad range of roles in regulating the transport activity of K^+^, ${\text{NO}}_{3}^{-}$, and ${\text{NH}}_{4}^{+}$ in Arabidopsis.

Besides, the CBL/CIPK-mediated phosphorylation pathways described above, other phosphorylation processes have been also reported to play a role in tuning the activity of K^+^ channels. For instance, the phosphatase AtPP2CA interacts with AtAKT2 and inhibits its activity (Chérel et al., 2002); a recent work identifies the direct binding of phosphatidic acid (PA) to two adjacent arginine residues in the ANK domain of OsAKT2 that leads to the inhibition of OsAKT2 activity (Shen et al., 2020).

In this work, we demonstrate through functional analysis in *Xenopus* oocytes that OsAKT2 of rice acts differently from a typical AKT2 channel. Further, the mutation analysis depicts a central role of K191 residue in controlling the rectification and voltage sensitivity of the channel. Using the Arabidopsis *akt2* mutant as a tool, we assemble and analyze the physiological role of OsAKT2.

## Results

### OsAKT2 functions mainly as an inward rectifier

**Figure 1:**
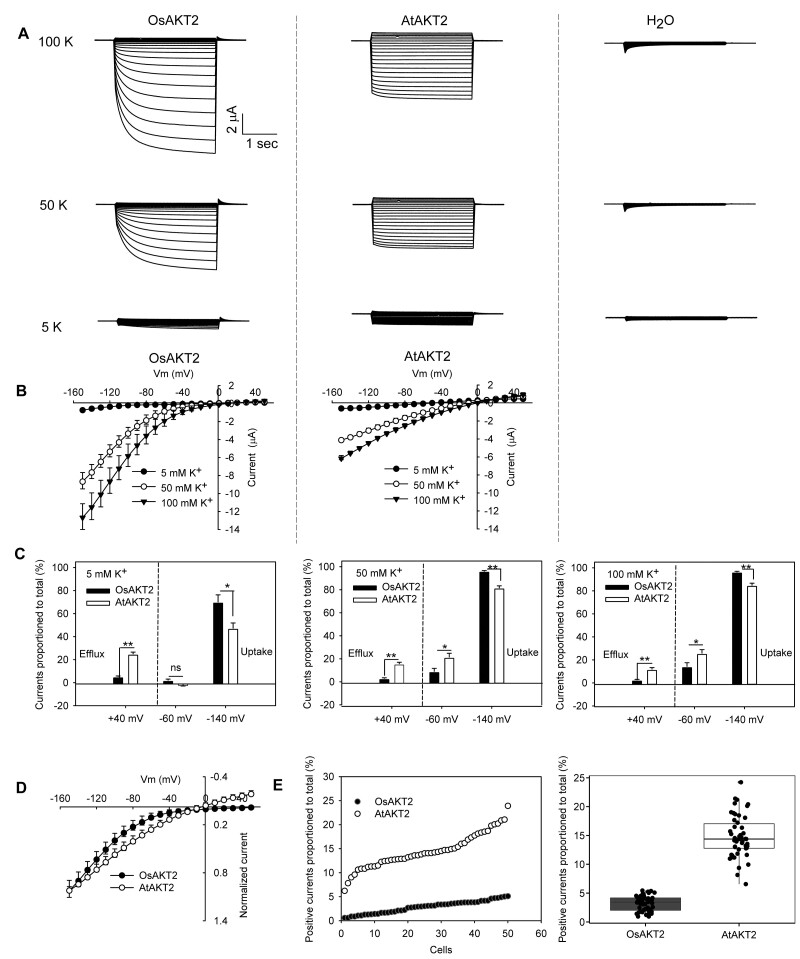
OsAKT2 functions distinctly from AtAKT2. A, Representative recordings for OsAKT2 and AtAKT2 in oocytes under 100, 50, or 5 mM K^+^ (pH 7.4). H_2_O-injected oocytes were used as controls. Stepped voltage pulses of 3 s ranging from −150 to +50 mV with 10 mV increments were imposed on the oocytes. The holding potential was 0 mV. B, Steady state currents were plotted against voltages imposed. C, Positive (efflux) amplitudes measured at +40 mV and negative (uptake) currents measured at −60 mV or −140 mV were proportioned to the total currents, respectively. Total currents used here were sums of maximal amplitudes of negative (absolute values) and positive currents. Data were means ± se. Significances were analyzed by Student’s *t* tests and expressed as *P < 0.05 and **P < 0.01, respectively. D, Currents recorded under 50 mM K^+^ (pH 7.4) were, respectively, normalized to their maximal currents measured at −150 mV to obtain a close comparison at the same scale between OsAKT2 and AtAKT2. E, Proportions of positive currents (measured at +40 mV) to total currents (sums of maximal currents of both directions) obtained from 50 randomly selected oocytes were scattered and queued for AtAKT2 and OsAKT2 (left). The right panel showed a statistical summary of 50 cells tested. In boxplot: center line, median; box limits, upper and lower quartiles; whiskers, 1.5× interquartile range; points, outliers.

The full-length sequence of OsAKT2 was determined according to our previous report to the Genebank (accession number: AFC96958) and was also consistent with a recent report (Shen et al., 2020). We noticed a shorter version of OsAKT2 (LOC_Os05g35410) annotated to the Rice Genome Annotation Database (http://rice.plantbiology.msu.edu) by lacking the first 152 amino acids at the N-terminus and was not successful to record current in *Xenopus* oocytes. The full-length OsAKT2 (Genebank accession number: AFC96958) encodes an ANK-containing Shaker K^+^ channel composed of 855 amino acids and shows the highest similarity to AtAKT2 (56%), followed by AtAKT1 (40%) and OsAKT1 (38%). In order to introduce a parallel comparison, the well-characterized AtAKT2 was included in the electrophysiological experiments in oocytes. Unexpectedly, under our experimental conditions with external K^+^ concentrations ranging from 5 to 100 mM, OsAKT2 behaved differently from a standard leak-like (bi-directional) AKT2 channel represented by AtAKT2 (Figure 1A). Further analysis of the current–voltage relationship indicated that OsAKT2 mediated mainly inward K^+^ uptake currents of large amplitude that showed clear voltage dependency. The outward components identified for a typical leak-like AKT2 channel were actually suppressed (Figure 1B). The increase in K^+^ concentration resulted in consistent augmentation of the inward amplitudes through OsAKT2, whereas AtAKT2 currents were simultaneously magnified in both directions and exhibited greatly reduced voltage dependencies (Figure 1B). At +40 mV, typical outward (efflux) currents were activated with AtAKT2, and the proportion of the positive amplitudes accounted ∼12–25% to the total currents (positive current to total current [P2T]) under 5, 50, or 100 mM K^+^ conditions; but almost no significant positive current (P2T < 5%) was measured with OsAKT2 (Figure 1C). At −60 mV or more hyperpolarized membrane potentials (for instance, −140 mV), the inward K^+^ uptake currents were dominant with both channels (Figure 1C). Since at a given K^+^ concentration/voltage, the current amplitude of OsAKT2 was obviously greater than AtAKT2, the current/voltage behaviors under 50 mM K^+^ were then, respectively, normalized to their maximal currents measured at −150 mV for further comparison at the same scale, which showed the absence of the outward components and strong voltage dependency of the OsAKT2 currents (Figure 1D). Under 50 mM K^+^, the proportions of positive currents measured at +40 mV to the total currents (sums of maximal currents of both directions) from 50 randomly selected oocytes were plotted and queued, showing statistically, ∼14.7% for AtAKT2 and <3.2% for OsAKT2 (Figure 1E).

**Figure 2:**
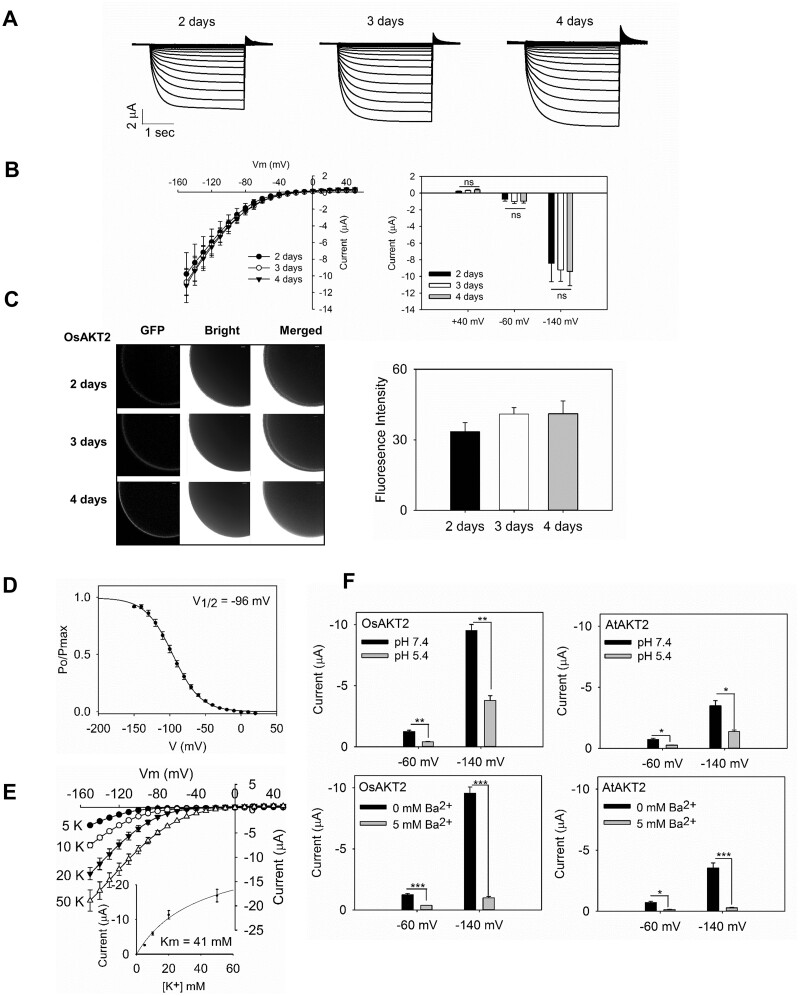
The expression of OsAKT2 in oocytes resembles an inward rectifier. Oocytes were recorded at different days after injection to elucidate the impacts of expression levels on the channel’s properties. A, Representative recordings at 2, 3, and 4 d of injection in a bath solution containing 50 mM K^+^, pH 7.4. B, The current–voltage relationship was summarized (left) and statistically analyzed at +40, −60, and −140 mV (right). Data represented means ± se from six cells. Significances were analyzed by Student’s *t* test. ns, not significant. C, The representative confocal microscopic images (left) of green fluorescence captured from oocytes injected with the eGFP fused OsAKT2 at 2, 3, and 4 d, Bar = 50 µm. The green fluorescence signals on the oocyte membrane were captured and quantified (right) using a Zeiss LSM710 confocal laser-scanning microscope incorporated with the Zen 2009 Light Edition software. Data represented means ± se (*n* > 15 cells). The significance was determined by one-way analysis of variance (ANOVA) with Duncan’s multiple range tests. D, The relative open probability (Po/Pmax) of OsAKT2 in 50 mM K^+^, pH 7.4. The half-maximal activation voltages (V_1/2_) were obtained from a Boltzmann fitting (solid line) of the data, using the following function: *P*_o_*/P*_max_ = [1/*Zg* (1 + exp ((*V-V_1/2_*) *F*/*RT*)], where F, R, and T represent Faraday’s constant, ideal gas constant, and absolute temperature, respectively, Zg is the equivalent gating charge, and *V*_1/2_ is the half-activation voltage, at which the current was half-activated. E, Currents in relation to membrane voltages recorded under varied K^+^ concentrations. Inset, a *Michaelis-menten* fitting of the current/concentration responses measured at −140 mV. F, Consistent properties between OsAKT2 and AtAKT2 with pH sensitivity and Ba^2+^ blockade. Upper, Currents measured at different membrane potentials (−60 and −140 mV) under 50 mM K^+^ for OsAKT2 (left) and AtAKT2 (right) in pH 7.4 (black) or pH 5.4 (gray) baths. Lower panels, Sensitivity to Ba^2+^ (5 mM). Currents were recorded in 50 mM K^+^ baths (pH 7.4). Data were means ± se (*n* = 4). *P < 0.05, **P < 0.01 (Student’s *t* test).

Because the Arabidopsis AtAKT2 engaged a transition between the inwardly rectified Mode 1 to the bi-directional voltage-independent Mode 2 upon phosphorylation status (Michard et al., 2005a, 2005b) and/or the accumulation of expression abundance (Dreyer et al., 2001). We particularly tested the current appearance of OsAKT2 in relation to levels of expression (days of oocyte injection). Under our conditions, large inward currents maximized >10 µA were recorded with OsAKT2-expressing oocytes 2 d after injection, and the currents were slightly developed along extended time of expression and saturated at Day 3 (Figure 2, A and B). In particular, the activation of outward and leak-like components was not apparently observed at the time course of expression (Figure 2, A and B). Furthermore, OsAKT2 was clearly detected on the plasma membrane of oocytes by the analysis of GFP fluorescence tagged to the protein. The abundance of OsAKT2 proteins increased along the time course of the expression and saturated at Day 3 (Figure 2C). Based on these observations and to avoid damages to oocytes with extended-expression time that compromise the accuracy of the recordings, we used uniformly the oocytes for current measurements 3 d of injection, at this time point, under our experimental conditions the property of OsAKT2 maintained not affected by the expression level. As depicted from the gating kinetics, the inward currents of OsAKT2 showed clear voltage dependency similar to a voltage-gated inward rectifier (Figure 2D). The concentration response of OsAKT2 showed a typical low-affinity property (Km 41 mM) described for an inward rectifier (Figure 2E). These data proved the evidence that OsAKT2 behaves distinctly as an inward rectifier in oocytes. Additionally, OsAKT2 showed consistent properties with AtAKT2, for the sensitivity to the blockade by Ba^2+^ and the strong inhibition by acidic pH (Figure 2F).

### A unique K191 residue plays a central role in the rectification control of OsAKT2

**Figure 3:**
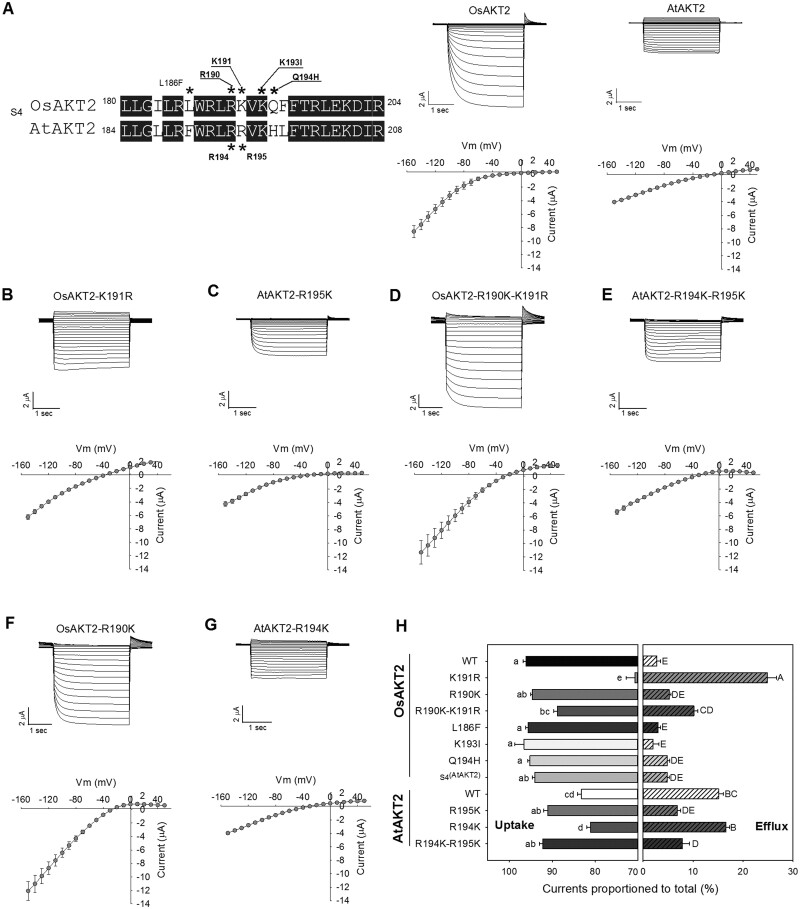
Determination of the involvement of key residues within the S4 segments in the rectification control between OsAKT2 and AtAKT2. A (left), Amino acid sequence alignment focused on the S4 segments between OsAKT2 and AtAKT2 channels. Residues marked with asterisks were tested by pair-wise substitution. A (right), and B–G, Representative recordings (upper) and currents–voltage relationships (lower) of tested point mutations. The experimental solutions contained 50 mM K^+^, pH 7.4. H, A statistical summary indicated proportions of positive (measured at +40 mV) or negative (measured at −140 mV) currents to the total currents of all point mutations or substitutions tested. Data were means ± se from at least four oocytes. Letters indicated significances at *P* < 0.05 determined by one-way ANOVA with Duncan’s multiple range tests.

Because in Shaker K^+^ channels, the S4 segment and the pore motif play important roles in voltage sensing and gating (Hoth et al., 2001; Sato et al., 2003; Pless et al., 2011; Clark et al., 2020), we made motif substitution and point mutations between OsAKT2 and AtATK2 to assess their involvement in the rectification control and voltage dependency of OsAKT2. To simplify the analysis, subsequent experiments were carried out under 50 mM K^+^, pH 7.4. Substitution of the entire pore motif from AtAKT2, which did not shift the reading frame or change the downstream amino acids sequence, resulted in loss-of-function of OsAKT2 in *Xenopus* oocytes (*n* > 40 cells; Supplemental Figure S1F). The substitution with the S4 segment of AtAKT2 did not affect the rectification or current amplitude of OsAKT2 (Figure 3H;Supplemental Figure S1A), but the opposite substitution abolished the function of AtAKT2 in oocytes (Supplemental Figure S1E).

Next, we focused on the alignment of the S4 sequences between OsAKT2 and AtAKT2 to further identify inconsistent residues, including the total charges (Figure 3A). The S4 of OsAKT2 contains one less positive charge than AtAKT2, balancing this positive charge by Q194H mutation did not introduce substantial change in the rectification or amplitude of OsAKT2 (Supplemental Figure S1B; Figure 3H), indicating the rectification difference between OsAKT2 and AtAKT2 may not be related to charges of the S4 segments. An apparent difference of charged residues’ arrayal between the two AKT2 channels was that two continuous RR (R194 and R195) in AtAKT2, while an RK (R190 and K191) arrayal was present to OsAKT2 (Figure 3A). Thus we generated sets of single or double mutations to these two residues, respectively, on either of the channels. Interestingly, the substitution of K191 by R (to mimick two continuous RR as in AtAKT2) readily restored the outward components and converted OsAKT2 to a typical weakly voltage-dependent, two-directional channel (Figure 3B). Under 50 mM K^+^, at +40 mV, the proportion of P2T was 24.8% with OsAKT2-K191R, even greater than that of AtAKT2 (P2T, 15.1%) (Figure 3H). Meanwhile, we observed a slight reduction of the inward current with OsAKT2-K191R (compare I/V curves of Figure 3B with Figure 1B, under 50 mM K^+^). Parallelly, the opposite mutation of the corresponding R195K (to RK arrayal as in OsAKT2) greatly diminished the outward current of AtAKT2 (P2T, 6.8%) and reshaped the voltage dependency of the channel similar to the inward rectifier-like OsAKT2 (Figure 3C). In addition, OsAKT2-K191R was inhibited by acidic pH and Ba^2+^ with both directions of currents, similar to that of AtAKT2 (Supplemental Figure 2; Figure 2F). These results indicated that the unique K191 residue attributes to the rectification control of OsAKT2 and retains the channel as an inward rectifier. Such control to a great extent also applies to the AtAKT2 channel. To further elucidate whether the rectification control by K191 was dominant or independent of the neighboring R, we tested more arrayal combinations centered with this site. The significantly increased outward current was also recorded with the double mutation OsAKT2-R190K-K191R (represent the KR arrayal), but with a weaker extent compared to K191R alone (P2T, 10.2% versus 24.8%, Figure 3D). The corresponding KR arrayal with AtAKT2 (AtAKT2-R194K) resulted in no effect on the rectification (P2T, 16.0%) or current amplitude (Figure 3G). For the KK arrayal, OsAKT2-R190K showed generally no difference from the wild-type OsAKT2 (P2T, 5.1%; Figure 3F). For the KK arrayal of AtAKT2, the AtAKT2-R194K-R195K double mutant behaved similarly as the R195K single mutant and mediated mainly inwardly rectified K^+^ uptake (P2T, 7.8%; Figure 3E).

We also tested the effect of other amino acids in the S4 region on the rectification of OsAKT2. For example, we verified the reported K193I (Michard et al., 2005b) (corresponding to the K197 site of AtAKT2), which only resulted in a dramatic change in the voltage sensitivity but had no effect on evoking the outward activity of OsAKT2 (Supplemental Figure S1D). Another L186F mutant was tested with no influence on the activity or rectification of OsAKT2 (Supplemental Figure S1C). The effects of mutations on both inward and outward activities of OsAKT2 and AtAKT2 were expressed as proportions to their respective total currents and summarized in Figure 3H. Taken together, these sets of analyses indicated a central role of the K191 residue in controlling the rectification/voltage-dependency of OsAKT2.

### OsAKT2 can be modulated by OsCBL1/OsCIPK23

**Figure 4:**
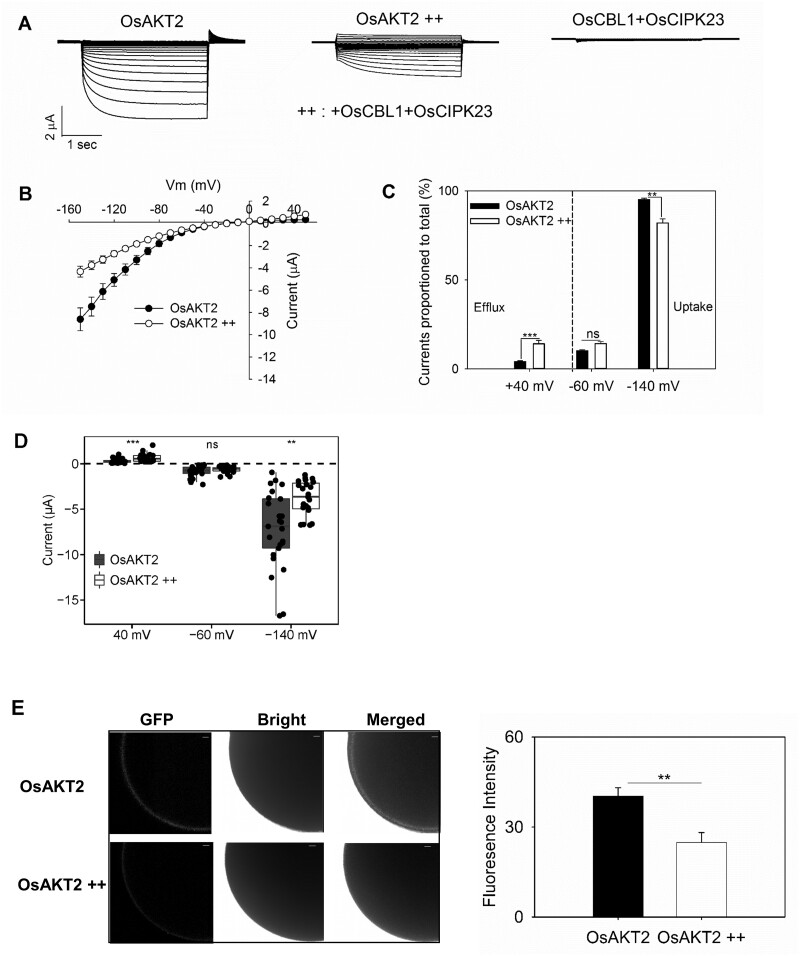
Co-expression of OsCBL1/OsCIPK23 causes inhibition to the K^+^ uptake current but activates the K^+^ efflux activity of OsAKT2 in oocytes. A, Representative recordings from oocytes expressing OsAKT2 alone (left), co-expressing OsAKT2 together with OsCBL1 and OsCIPK23 cRNAs (OsAKT2++, middle), or injected with OsCBL1 and OsCIPK23 cRNAs in the absence of OsAKT2 (right). The testing conditions were 50 mM K^+^, pH 7.4. B, Currents–voltage relationships of OsAKT2 (closed circles) and OsAKT2++ (open circles). C, Proportions of positive (at +40 mV) or negative (at −60 mV or −140 mV) currents to the total amplitude of OsAKT2 (black bars) and OsAKT2++ (white bars). D, The distribution of positive (at +40 mV), resting (at −60 mV), and hyperpolarized (at −140 mV) currents. Data were means ± se from 24 oocytes. E, The representative confocal microscopic images (left) of green fluorescence from oocytes injected with OsAKT2-GFP or OsAKT2^++^-GFP, Bar = 50 µm. Relative GFP fluorescence intensity (right) was measured at the membrane of the oocyte and statistically analyzed. Data represented means ± se (*n* > 15 cells). **P < 0.01, ***P < 0.001 by Student’s *t* test, NS denoted nonsignificant difference.

Many Shaker K^+^ channels are regulated by the CBL/CIPK pathway (Xu et al., 2006; Ho et al., 2009; Wu et al., 2011; Li et al., 2014; Ragel et al., 2015; Straub et al., 2017; Sánchez-Barrena et al., 2020; Wang et al., 2020). Since OsAKT2 also contains the required ANK domains, we thus tested the possibility of such regulation. This was realized through the co-expression in oocytes by injection of both OsCBL1 and OsCIPK23 (Li et al., 2014) together with OsAKT2 (OsAKT2++). Oocytes injected only with OsCBL1 and OsCIPK23 (in the absence of OsAKT2) were used for negative controls, indicating that the expression of the kinase genes did not activate the endogenous activity in oocytes (Figure 4A, right). Compared to OsAKT2 alone (Figure 4A, left), the co-expression of OsCBL1 and OsCIPK23 (Figure 4A, middle) resulted in up to half-way (49.6%) reduction of the inward current of OsAKT2 which was accompanied by significant recruitment of the outward current (+100.6%, Figure 4, B and C). This phenomenon was repeatedly observed (Figure 4D, *n* = 24). The inhibition of the inward activity to a certain extent was consistent with the reduction of the abundance of the channel protein targeted to the oocyte membrane as observed by the GFP-tagged fluorescence (Figure 4E).

**Figure 5:**
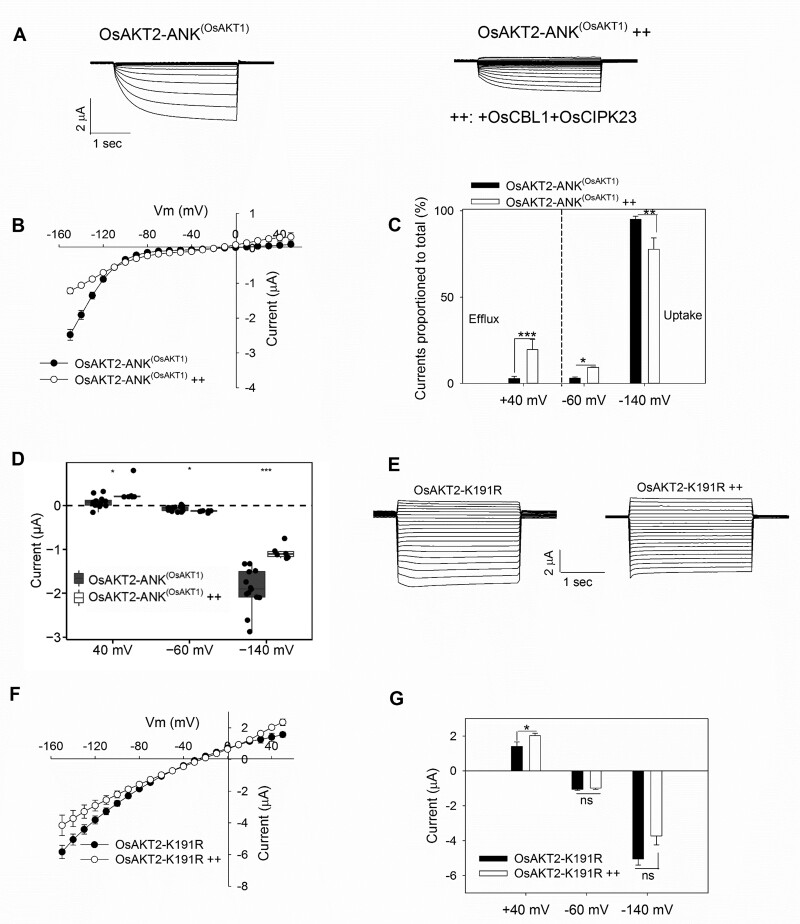
The OsCBL1/OsCIPK23 modulation also acts on OsAKT2 substituted by the ANK domain from OsAKT1 and the OsAKT2-K191R mutant channel. A–D, OsAKT2 with the ANK domain substituted from OsAKT1 (OsAKT2-ANK^(OsAKT1)^) was modulated similarly as the wild-type OsAKT2 by OsCBL1/OsCIPK23 (*n* > 6). Data presentation was in the same order as described in Figure 4, A–D. E, Representative recordings and F, showing the reduction of inward current and further activation of the outward activity through the modulation by OsCBL1/OsCIPK23 (OsAKT2-K191R++). G, Statistical summary of currents determined at +40, −60, or −140 mV (*n* > 6). Oocytes were recorded in bath solutions containing 50 mM K^+^, pH 7.4. Data were means ± se. *Significance at *P* < 0.05 (Student’s *t* test).

Taken into account that the specificity of the physical interaction between the CIPK kinase and target K^+^ channel proteins is determined by the ANK repeat domains (Sánchez-Barrena et al., 2020) and that the OsAKT1 channel can be also modulated by the same OsCBL1/OsCIPK23 complex (Li et al., 2014), we substituted the ANK domain of OsAKT2 by the corresponding domain of OsAKT1 based on a sequence alignment of the ANK domains among several K^+^ channels (Supplemental Figure S3). The resultant OsAKT2-ANK^(OsAKT1)^, though greatly reduced in the current amplitude (Figure 5, A and B), was regulated similarly (compared to the wild-type OsAKT2, Figure 4) by OsCBL1/OsCIPK23 (Figure 5, A–C). Figure 5D listed a statistical summary of all related recordings (*n* > 6), indicating the consistency with the repeated observations from the wild-type OsAKT2 (see Figure 4). Unfortunately, our attempts for a reverse substitution with OsAKT1-ANK^(OsAKT2)^ alone or co-expressed with OsCBL1 and OsCIPK23 were not successful, due to no measurable currents that occurred in oocytes. These results imply the presence of similar binding sites within the ANK domains between OsAKT2 and OsAKT1 (e.g. the repeat two with the highest similarity; Supplemental Figure S3) is responsible for the interaction with the OsCBL1/OsCIPK23 complex. Furthermore, the OsAKT2-K191R was similarly regulated by OsCBL1/OsCIPK23, resulting in a slight reduction of the inward current and a more strengthened augmentation of the outward current (Figure 5, E–G). In either case with the wild-type OsAKT2++ (Figure 4B), OsAKT2-ANK^(OsAKT1)^++ (Figure 5B), or OsAKT2-K191R++ (Figure 5F), the modulation by OsCBL1/OsCIPK23 seemed to have little effect on the voltage-dependency of the channels. Therefore, in particular respect to the functional “defect” of OsAKT2’s outward activity, the modulation through OsCBL1/OsCIPK23 may partly represent a complementation or rescue mechanism.

### OsAKT2 complements the *akt2* mutant plants with elevated vascular K^+^ but does not overcome the sugar shortage in phloem

**Figure 6:**
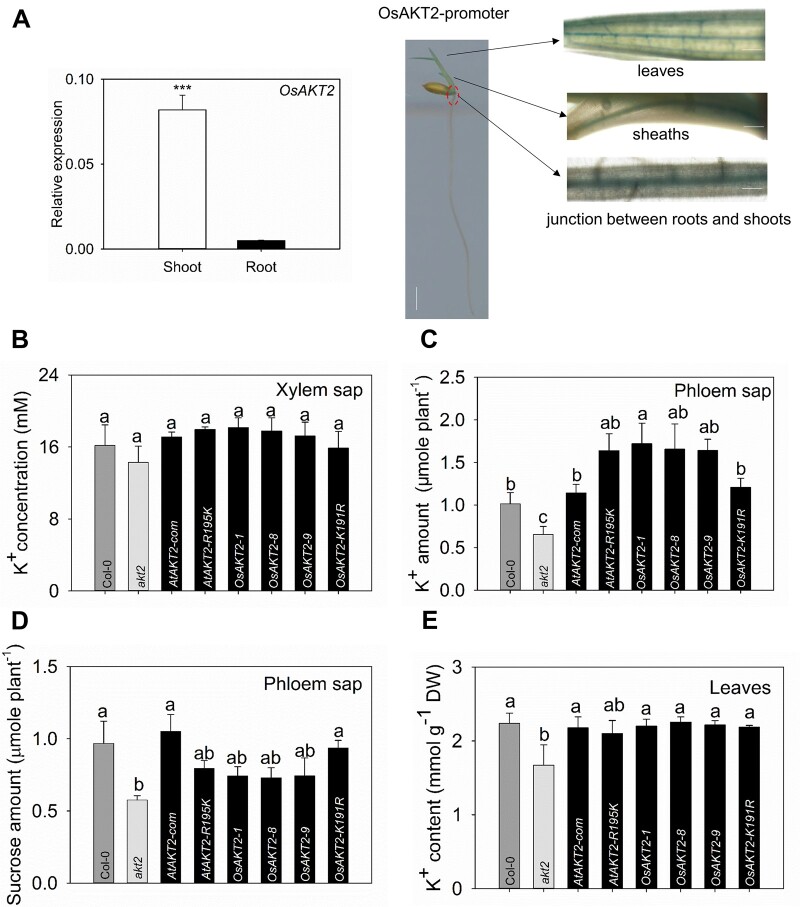
Physiological assessment of OsAKT2 in comparison to AtAKT2 and mutated AKT2 channels by the OE in the *akt2* mutant Arabidopsis plants. A, OsAKT2 was mainly expressed in the vascular tissue of the aerial parts. Gene expression profiles were determined with rice (cv *Nipponbare*) seedlings under hydroponic growth conditions. Relative expression abundances (left), respectively, in shoots and roots of OsAKT2 genes were evaluated by real-time quantitative RT-qPCR analysis under 1 mM K^+^. A genomic fragment of 2,576-bp upstream the ATG start codon of OsAKT2 gene, was fused to the GUS reporter gene constructed in pCAMBIA1301 plasmid and transformed into *Nipponbare* rice via callus transformation. The promoter-GUS reporter gene activity (right) assayed in 5-d-old seedlings was localized to the vascular tissues of major veins of leaves (upper), sheaths (middle), and the junction between roots and shoots (lower).Vertical line represents bar = 0.5 cm, horizontal line represents bar = 10  m. B–E, The phenotype was observed from 7-week-old Arabidopsis seedlings grown under 1 mM K^+^ irrigated soil matrix. Three T3 homozygous-independent OE lines (*OsAKT2-1*, *OsAKT2-8*, and *OsAKT2-9*), *AtAKT2* complementation line (*AtAKT2-com*), *AtAKT2-R195K* lines, and *OsAKT2-K191R* lines (T2 heterozygotes, each seedling was verified by PCR before the experiments) were tested in comparison to the *akt2* mutant and the wild-type Col-0 plants. B, K^+^ concentrations in the xylem sap. The amounts of K^+^ (C) and sucrose (D) amount in the phloem sap (collected during 2 h, in 20 mM EDTA buffer) were expressed as micromole per plant, respectively. E, K^+^ content of plant leaves (*n* = 15). Data were means ± se. Statistical significance analysis was performed by one-way ANOVA with Duncan’s multiple range test at *P* < 0.05.

OsAKT2 was expressed mainly in shoots and weakly in roots as measured by reverse transcription-quantitative PCR (RT-qPCR; Figure 6A, left). Furthermore, the promoter-GUS assays showed that in shoots OsAKT2 expression was concentrated in the vascular bundles of leaves, sheaths, and the junction between roots and shoots (Figure 6A, right). This localization profile was consistent with the recent report (Shen et al., 2020). To simplify the physiological analysis, we used the well-defined Arabidopsis *akt2* mutant (Dennison et al., 2001; Gajdanowicz et al., 2011) as a tool to assemble the physiological relevance of OsAKT2 in particular relation to the rectification diversity.

K^+^ concentrations in the xylem sap among *akt2* mutant, the wild-type plant, and different transgenic lines did not show a significant difference (Figure 6B). Consistent with previous reports (Deeken et al., 2002), knockout of the AKT2 function resulted in 35% reduced K^+^ in the phloem sap of *akt2* mutant plants, whereas overexpression (OE) of all of the constructs overcame the defect of phloem K^+^ of the mutant (Figure 6C). The complementation by the wild-type AtAKT2 channel resulted in 8% increased phloem K^+^ comparable to the wild-type plant. The expression of the *AtAKT2-R195K* mutation channel obtained a further increment of phloem K^+^ by 15%, while the three *OsAKT2* OE lines behaved similarly as *AtAKT2-R195K* in elevating the phloem K^+^ and exhibit superior to the wild-type plant. The *OsAKT2-K191R* contributed, to a smaller extent, to the restoration of phloem K^+^, an effect mimicking the wild-type *AtAKT2* (Figure 6C). Interestingly, the contributions of the transgenes on the phloem sucrose displayed oppositely to the effects on K^+^: the inward-likes including OsAKT2 and AtAKT2-R195K contributed mainly to the loading of K^+^ to the phloem, whereas those exhibiting bi-directional rectifications (the wild-type AtAKT2 and OsAKT2-K191R) emphasized an enhanced contribution to the loading of sucrose (Figure 6D). Nonetheless, OE of all constructs resulted in a significant rescue in leaf K^+^ content, depleted in the *akt2* mutant (Figure 6E). Thus these functional analyses proved further correlations between the rectification diversity and its physiological relevance in planta.

### OE of OsAKT2 enhances salt stress tolerance in Arabidopsis

**Figure 7:**
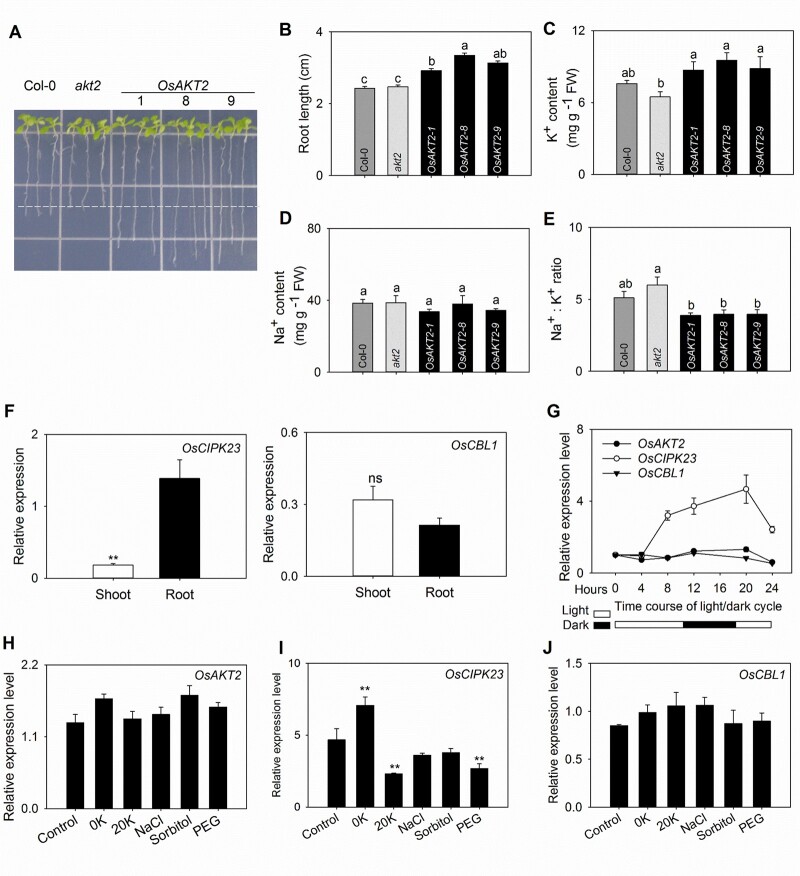
OE of OsAKT2 ameliorates the salt tolerance in Arabidopsis and the gene expression profiles of OsAKT2 and its modulating kinase genes OsCBL1 and OsCIPK23 in rice seedlings. A, Salt tolerance was assessed on OsAKT2-OE, *akt2*, and Col-0 Arabidopsis plants grown for 10 d on agar-solidified nutrient plates supplied with 100 mM NaCl. three individual lines (1, 8, and 9) of OsAKT2-OE plants were included. B, Principal root length. C–E, shoot K^+^ (C), Na^+^ (D) contents, and the Na^+^/K^+^ ratio (E) were determined, respectively, from 50 plants. Data were means ± se of three independent experiments. Statistical significances were analyzed by one-way ANOVA with Duncan’s multiple range tests. F–J, Gene expression profiles were determined with rice seedlings under hydroponic growth conditions. Rice (cv *Nipponbare*) seedlings of 7-d-old were subjected to hydroponic treatments under normal (1 mM K^+^) or frustrating conditions. Levels of gene expression were evaluated by real-time quantitative RT-qPCR analysis. F, Relative expression abundances, respectively, determined in shoots and roots of OsCIPK23 (left) and OsCBL1 (right) genes under nonstressed conditions (1 mM K^+^). G, Circadian dynamics of the expression of OsAKT2 (closed circles), OsCIPK23 (open circles), and OsCBL1 (closed triangles) genes during a day/night cycle of 24 h. The light and dark periods were indicated, respectively, by blank and solid bars below. Experiments were carried out under normal conditions (1 mM K^+^). Relative expression levels were determined at time points 0, 4, 8, 12, 20, or 24 h from the first sample at 14:00. H–J, Expression levels of OsAKT2 (H), OsCIPK23 (I), and OsCBL1 (J) in response to 20 h treatments with K^+^ depletion (0 K), K^+^ oversupply (2 0K, 20 mM K^+^), 100 mM NaCl (NaCl), 180 mM sorbitol (Sorbitol), or 15% PEG-6000 (PEG). The plants grown under 1 mM K^+^ (in IRRI nutrient solution) were used as controls. Gene expression abundance was quantized with 2^−ΔΔCt^ and normalized to the expression level of the OsActin gene. Data were means ± se from four individual experiments. *P < 0.05 and **P < 0.01.

Since plants’ tolerance to salt stress is closely related to their K^+^ transport and accumulation, based on the above observation that OsAKT2 strongly contributed to K^+^ transport in vascular tissues, we further assessed its capability of salt tolerance using Arabidopsis. Under plate experiments supplied with 100 mM NaCl, OE of *OsAKT2* (in the background of *akt2* mutant) dramatically overcame the sensitivity to salt (Figure 7A) with 26% increase in root length (Figure 7B). The shoots of the OE lines contained 34%–47% higher concentration of K^+^ than the *akt2* mutant plants (Figure 7C) that resulted in reduced Na^+^/K^+^ ratios (Figure 7E) though the accumulation of Na^+^ in shoots remained not significantly affected (Figure 7D). Therefore, OsAKT2 provides enhanced salt tolerance by facilitating K^+^ accumulation to shoots and reducing the Na^+^/K^+^ ratio that is important for salt tolerance (Fuchs et al., 2005). This result was consistent with the recent description that mutation of OsAKT2 impaired the salt tolerance in rice (Tian et al., 2021).

Because in oocytes, the function of OsAKT2 can be reshaped by protein kinases OsCBL1/OsCIPK23, and the activation of the kinase activity is often a consequence of environmental fluctuations, we then analyzed the expression profiles of the three genes particularly in response to changes in K^+^ availability and salt stresses in rice plant. Using RT-qPCR, we could locate the expression of OsAKT2 mainly in shoots and weakly in roots (Figure 6A), whereas OsCIPK23 expression was not only high in roots but also showed considerable expression in shoots. OsCBL1 expression was similar in shoots and roots (Figure 7F). For the day–night cycles experiment, the light started at 8:00, and we took the first sample at 14:00 as 0  h. In shoots, under normal conditions (1  mM K^+^) without stress, the expression of OsCIPK23 (but not for OsAKT2 or OsCBL1) showed somehow a circadian dynamics over continuous observations during a time-course of 24  h corresponding to a shift in day–night cycles in the growth chamber (Figure 7G): the transcripts accumulated along the dark cycle (12–20  h) and peaked at the end of dark cycle (20  h), then rapidly fell to normal level upon the switch on of the light (20–24  h). Considered that the outward activity of OsAKT2 could be completed by OsCBL1/OsCIPK23 (Figures 4 and 5) and such outward activity facilitates the transport of photosynthetic sugars, the expression profile of OsCIPK23 (high in dark and low in light) is in line with the phenomenon that OsAKT2 is not capable of overcoming the sugar shortage in Arabidopsis (Figure 6D). In this sense, under normal conditions, OsAKT2 does not contribute to sugar transport.

Under varying K^+^ supplement (0, 1, or 20  mM), or stress conditions (100  mM NaCl, 180  mM sorbitol, or 15% polyethylene glycol (PEG)), the expression of OsAKT2 and OsCBL1 was generally not affected (Figure 7, H and J). Whereas OsCIPK23 was induced by K^+^ depletion and suppressed by high K^+^ supply or PEG mimicked drought stress treatments, but no influence by the NaCl and Sorbitol treatments (Figure 7I). Considering the half-way suppression of K^+^ uptake activity through the modulation by OsCBL1/OsCIPK23 (Figure 4), we speculate that OsAKT2 is more active under high K^+^ or abiotic stresses such as drought and salt.

## Discussion

### OsAKT2 is retained as an inward rectifier

In Arabidopsis, AKT2 represents a particular Shaker K^+^ channel that mediates bi-directional K^+^ fluxes in the weakly voltage-dependent manner and thus is also called a dual-rectifying, weakly rectifying, or “leak-like” K^+^ channel (Lacombe et al., 2000; Dreyer et al., 2017). To date, several more reports on such AKT2-type K^+^ channels have been achieved in plants: ZMK2 in *Zea mays* (Philippar et al., 1999), VFK1 in *Vicia faba* (Ache et al., 2001), PTK2 in *Populus tremula* (Langer et al., 2002), NKT2 in *Nicotiana tabacum* (Sano et al., 2007), HvAKT2 in *Hordeum vulgare* (Boscari et al., 2009), VvK3.1 in *Vitis vinifera* (Nieves-Cordones et al., 2019). In the present work based on repeated electrophysiological observations in oocytes, we find that the rice OsAKT2 acts rather as an inward rectifier that shows strong voltage-dependency, and the outward and/or the weakly voltage-dependent components were drastically suppressed (Figures 1 and 2). This “abnormal” phenomenon can be traced back to consistent indications (especially evaluated by the current/voltage relationships) from a more recent work on the same channel, in which a regulatory mechanism by PA is emphasized (Shen et al., 2020).

Next, we identify that a unique K191 residue located in the S4 transmembrane segment plays a central role in the rectification control of OsAKT2 (Figure 3). Here we define the term “rectification” as both the direction and the voltage-dependency of the currents. This site is distinct from almost all AKT2 channels reported, including the well-defined AtAKT2 (Supplemental Figure S4; Figure 3A). The dataset obtained by the mutations on this site that exchange the rectification between OsAKT2 and the Arabidopsis AtAKT2 (Figure 3) provides further support that the rectification distinction displayed by OsAKT2 is an intrinsic property of the channel, and suggests a common role of this site on the controlling of the rectification between at least the two channels. Concerning the mode of action of the K191 residue, one possibility lies in that the presence of this unique site may prevent the transition of the channel from Mode 1 (inward rectifier) to Mode 2 (bi-directional *K*_weak_) thus the channel is retained in Mode 1 and acts mainly as an inward rectifier (Michard et al., 2005a, 2005b). To this respect, it worths further investigation of how the K191 participates in the phosphorylation regulation of AKT2 channels? Additionally, the amino acid sequence of the barley HvAKT2 contains an identical K residue at this site (Supplemental Figure S4). According to the representative electrophysiological recording traces and statistical current–voltage relationship data (Boscari et al., 2009), HvAKT2 may potentially represent another exception of plant AKT2 channels. To this end, the commonality of the role of K191 residue (or equivalent positions) in controlling channels’ rectification may be further expanded.

A fundamental response of plants to environmental fluctuations is the initiation of signaling processes that often involve the activation of diverse protein kinases, including CBLs and CIPKs (Sanyal et al., 2016). In this work, we reveal that the expression of OsCIPK23 is induced under dark and by the K^+^ depletion (Figure 7). Furthermore, we find that OsAKT2 can be directly regulated by the OsCBL1/OsCIPK23 module (Figure 4) previously reported for OsAKT1 (Li et al., 2014). Such modulation, though suppressing the inward activity, results in partial restoration of the outward components of OsAKT2 (Figure 4). A recent crystallography study depicts that the ANK domain is necessary for the physical interaction with the CIPK23 complex (Sánchez-Barrena et al., 2020). The presence of similar ANK domain 2 between OsAKT2 and OsAKT1 (Supplemental Figure S4) allows modulation of OsAKT2 activity by the same OsCBL1/OsCIPK23 module, but with a consequence differed from that of OsAKT1 (Figure 5). Nevertheless, the CBL/CIPK modulation to a certain extent compensates the functional “defect” of OsAKT2 by completing the “missing” outward activity and makes the channel “more AKT2”. However, this may represent an alternative mechanism of functional completeness that is independent of the structural “locking” by the K191 residue, since the OsAKT2-K191R is similarly modulated by OsCBL1/OsCIPK23 (Figure 5).

### The physiological role of OsAKT2

In this work, we demonstrate in the Arabidopsis *akt2* mutant plant that the physiological role of OsAKT2 highlights a strong contribution to the translocation of K^+^ in shoot phloem with no significant facilitation to the loading of photoassimilates (Figure 6). Our gene expression analysis and promoter-GUS activity assays (Figure 6A) provide a highly consistent localization of OsAKT2 to the phloem tissues of the root–shoot junction section, sheaths, and leaf veins with recent reports (Shen et al., 2020). Assuming that the outward activity of AKT2 channels is the major contributor to the transport of sugars through the phloem pathway (Dennison et al., 2001; Deeken et al., 2002; Gajdanowicz et al., 2011; Dreyer et al., 2017; Ragel et al., 2019), the lack of pronounced outward components exhibited by OsAKT2 here would indeed compromise its involvement in the loading and transport of sugars. This notion has been further enhanced by our parallel analyses of AKT2 channels with changed rectifications in the same Arabidopsis *akt2* mutant system: the expression of the bi-directional AKT2s such as the wild-type AtAKT2 and OsAKT2-K191R complements not only the phloem K^+^ deficiency of the mutant plants but also rescues the shortage of sucrose in the phloem (Figure 6); Conversely, the physiological relevance of the inward-likes, including AtAKT2-R195K and the three independent wild-type OsAKT2 lines emphasizes a major role in mediating the loading of K^+^ to the phloem that probably reduces their facilitation to the transport of sucrose (Figure 6). Taken together, the physiological relevances in planta of the channels and their mutations with altered rectification are in fair agreement with their functional diversity characterized in oocytes (Figures 1–3). In addition, the impairment to sucrose transport in the phloem has also been observed under salt stress conditions by a more recent work with the *osakt2* mutant rice (Tian et al., 2021). Therefore, despite the high degrees of similarities to the typical AKT2 channels, OsAKT2 operates distinctly both in its functional aspect and physiological significance.

In conclusion, we demonstrate here an unusual “inward-mainly” function of OsAKT2 in oocytes that is originated from the presence of a unique K191 residue. Consistently the physiological relevance of OsAKT2 emphasizes the contribution to the loading of K^+^ to the phloem with probably reducing facilitation to the transport of sucrose.

## Materials and methods

### Expression in oocytes and two-electrode voltage-clamp experiments

The coding sequence of OsAKT2 (JN989970) was cloned and inserted into the oocyte expression vector pCI, the primers were listed in Supplemental Table S1. Oocytes were isolated from mature *Xenopus* frogs and prepared as previously reported (Su et al., 2005; Hao et al., 2016). The coding sequences of OsCBL1 and OsCIPK23 (Li et al., 2014) were cloned into pT7TS vector, and a mMESSAGE mMACHINE T7 Transcription Kit (Thermo) was used to generate polyadenylated cRNA. Oocytes were micro-injected (Nanoliter 2000) with 59.8  ng plasmid DNA containing the wild-type channel or mutations. For co-expression, 29.9  ng cRNA of each kinase was injected together with the channel DNA (in pCI plasmid). Oocytes injected with the same amounts of H_2_O or kinase cRNAs only were used as controls, then incubated at 19°C in ND96 solution (in millimolar, 96 NaCl, 2 KCl, 1.8 CaCl_2_, 1 MgCl_2_, 5 HEPES–NaOH, and pH 7.4). Oocyte currents were detected using the two-electrode voltage-clamp after 3 d. The bath solution contained (in millimolar): 1.8 CaCl_2_, 1 MgCl_2_, 5 HEPES–NaOH, and pH 7.4. Desired concentrations of KCl and NaCl were used to adjust the constant ionic strength in the solution.

### Microscopic analysis of fluorescent intensity in oocytes

An enhanced green fluorescence protein (eGFP) was fused to the C-termini of the channel proteins. Upon the injection and expression in oocytes as described above, the green fluorescence signals on the plasma membrane of the oocytes were observed and imaged using a Zeiss LSM710 confocal laser-scanning microscope (Zeiss, Oberkochen, Germany). eGFP was excited at 488 nm (Digital Gain: 1.0, Scan time: 3.87 s). Because of the large volumes of the oocytes, only partial globes of the cells could be visualized (10× magnification). The eGFP fluorescence intensity was measured and statistically analyzed using the Zen 2009 Light Edition software of the instrument.

### Transgenic plant materials and growth conditions

Arabidopsis ecotype Columbia (Col-0) was used as the wild-type plant. The homozygous *akt2* mutants (SALK-017212) were obtained from the Arabidopsis Biological Resource Center. The coding sequence of OsAKT2 (AtAKT2, AtAKT2-R195K, and OsAKT2-K191R) was inserted into the pCAMBIA1301-35S-NOS vector, then transformed into *akt2* mutant plants. The independent transgenic plants *OsAKT2-1, OsAKT2-8*, and *OsAKT2-9*, *AtAKT2-com*, *AtAKT2-R195K*, and *OsAKT2-K191R* were used for experiments. Hydroponic culture solution according to previously described (Han et al., 2015). The growth chamber was set with 12 h/12 h light/dark. (1) For the pot culture experiment, Arabidopsis seedlings were transferred to the flowerpots containing a mixture of soil, perlite, and vermiculite (2:1:1, v/v/v) (solidified matrix cleaned 5 times), then treated with hydroponic culture solution noted above, and changed twice a week. (2) For salt stress plate treatments, seeds germinated on nutrient plates containing 1/4 Hydroponic culture medium. Five days later, Seeds of uniform growth were moved to 100-mM NaCl medium which other elements remain constant. The samples were harvested after 10 d for the measurement of root length and elemental concentrations. Plant K^+^ and Na^+^ concentrations were measured by flame photometer.

### Measurement of K^+^ and sucrose content

Seven-week-old Arabidopsis plant shoots were excised. Then the xylem sap was collected using glass capillaries (Han et al., 2016). The phloem sap was infiltrated in 20 mM EDTA buffer for 2 h from uniformly excised shoots (Beneteau et al., 2010; Killiny, 2019). Sucrose concentrations in the collected solution were determined by a sucrose content assay kit. K^+^ in the saps and leaves were measured by flame photometer.

### Gene expression analysis in rice plants

Rice Nipponbare seedlings were grown in a modified international rice research institute (IRRI) solution (Yang et al., 2015) under a 16/8 h photoperiod in a growth chamber. Ten-day-old seedlings were exposed to different treatments for 24 h. Total RNAs were extracted with Trizol Reagent (Han et al., 2015). The RT-qPCR used the SYBR Mix (Vazyme, Nanjing, China) and LightCycler 480 Instrument (Roche, Basel, Switzerland), the primers were listed in Supplemental Table S1.

### Accession numbers

Sequence data for this article can be found in the GenBank data libraries or the Arabidopsis Information Resource under accession numbers: AFC96958 (OsAKT2) and AT4G22200 (AtAKT2).

## Supplemental data 

The following materials are available in the online version of this article.

**Supplemental Figure S1.** Effects of substitutions or point mutations that do not affect the rectification of OsAKT2 and AtAKT2.

**Supplemental Figure S2.** Effect of external pH and Ba^2+^ on OsAKT2-K191R.

**Supplemental Figure S3.** Amino acid sequence alignment of the ANK domains among AKT2 and AKT1 channels.

**Supplemental Figure S4.** S4 amino acid sequence comparison among known AKT2 channels.

**Supplemental Table S1.** Primer sequences used in this study.

## Supplementary Material

kiab462_Supplementary_Data

## Notes

Y.H.S. conceived the research. Y.N.H. did the experiments and analyzed the data. S.Y.Y. contributed to the cloning and construction of OsAKT2 and kinase genes. J.L.L. contributed to the preliminary electrophysiological experiments. S.F.W. and J.J.W. participated in the gene expression experiments. D.L.H. assisted in electrophysiological experiments. Y.H.S. and Y.N.H. wrote the paper.

The author responsible for distribution of materials integral to the findings presented in this article in accordance with the policy described in the Instructions for Authors (https://academic.oup.com/plphys/pages/general-instructions) is Yan-Hua Su (yhsu@issas.ac.cn).
