# Supplementary material for: The rectification control and physiological relevance of potassium channel OsAKT2
Source: Plant Physiol. 2021 Oct 2;187(4):2296–310. doi: 10.1093/plphys/kiab462 (PMC8644434; doi:10.1093/plphys/kiab462)
Supplement: kiab462_Supplementary_Data [file kiab462_supplementary_data.pdf]

Supplemental Data

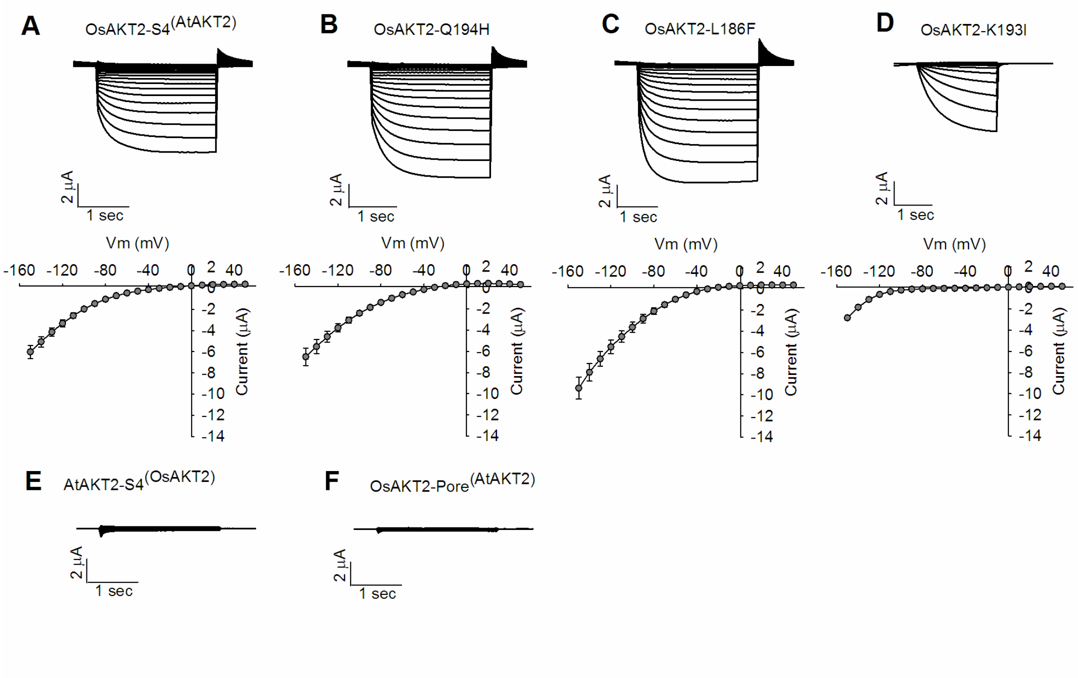

Supplemental Figure S1. Effects of substitutions or point mutations that do not affect the rectification of OsAKT2 and AtAKT2.

Representative recordings (upper panels) and currents-voltage relationships (lower panels) of the AKT2 mutants measured in 50 mM K^+, pH 7.4: A, OsAKT2-S4$^{(AtAKT2)}$, B, OsAKT2-Q194H, C, OsAKT2-L186F, D, AtAKT2-K193I. E, The intact substitution of S4 AtAKT2-S4$^{(OsAKT2)}$ or F, pore motif OsAKT2-Pore$^{(AtAKT2)}$ caused no detectable current with AtKAT2. Data were means ± SE (n = 4 - 8).

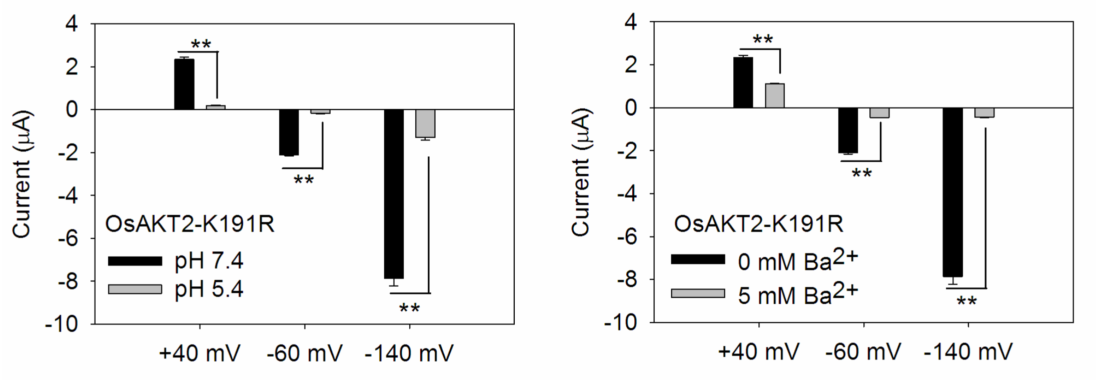

Supplemental Figure S2. Effect of external pH and Ba^{2+} on OsAKT2-K191R. Currents measured at different membrane potentials (+40, -60 and -140 mV) under 50 mM K^+ for OsAKT2-K191R in pH 7.4 (black) or pH 5.4 (grey) baths (left). Sensitivity to Ba^{2+} (5 mM, right). Currents were recorded in 50 mM K^+ baths (pH 7.4). Data were means ± SE (n = 4). *$p < 0.05$, **$p < 0.01$ (Student's *t*-test).

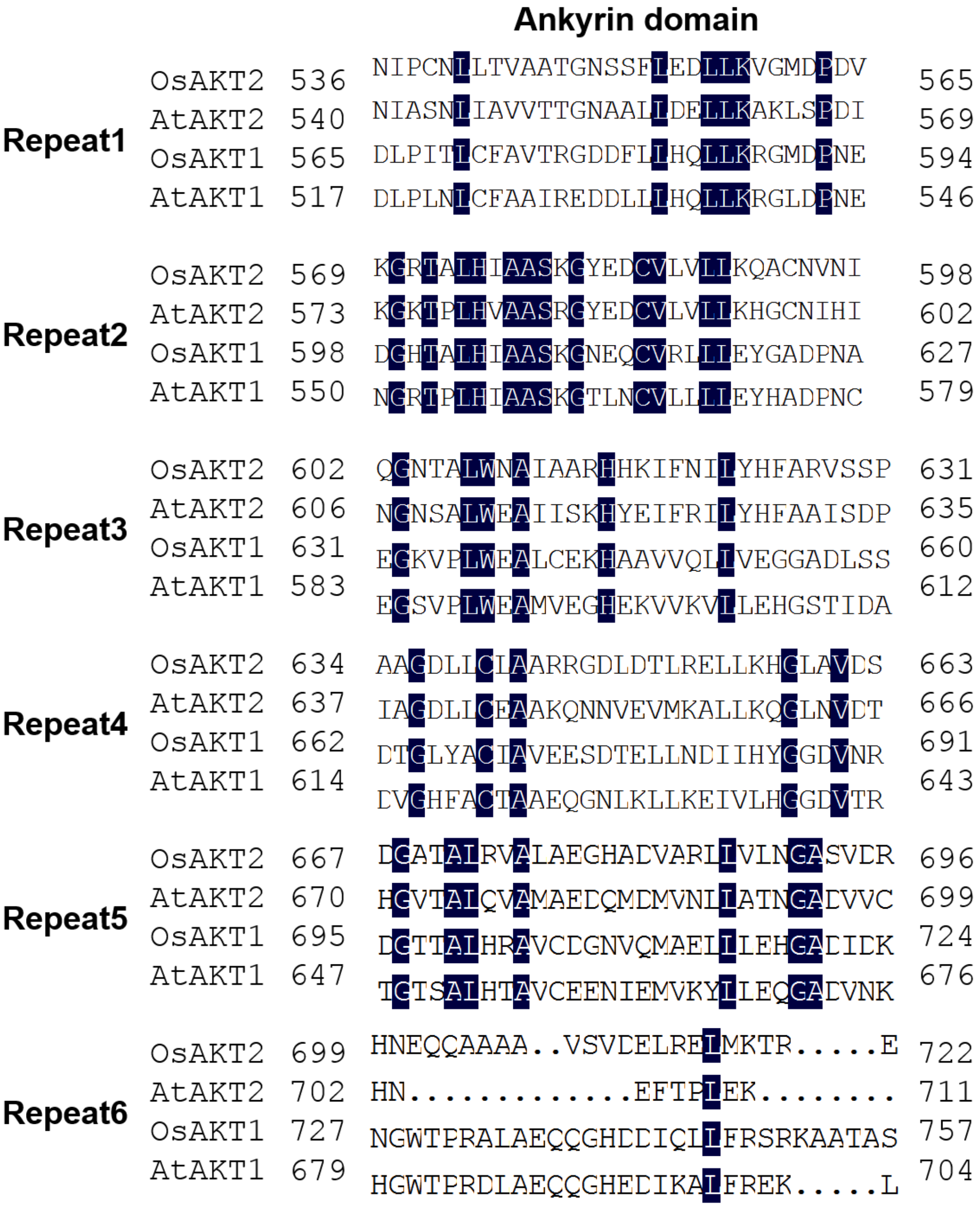

Supplemental Figure S3. Amino acid sequence alignment of the ANK domains among AKT2 and AKT1 channels.

The sequences were aligned using DNAMAN software. The access numbers of aligned sequences were: AtAKT1 (NP_180233), OsAKT1 (AK120308), OsAKT2 (JN989970) and AtAKT2 (AAA97865). Conserved amino acids were highlighted in black.

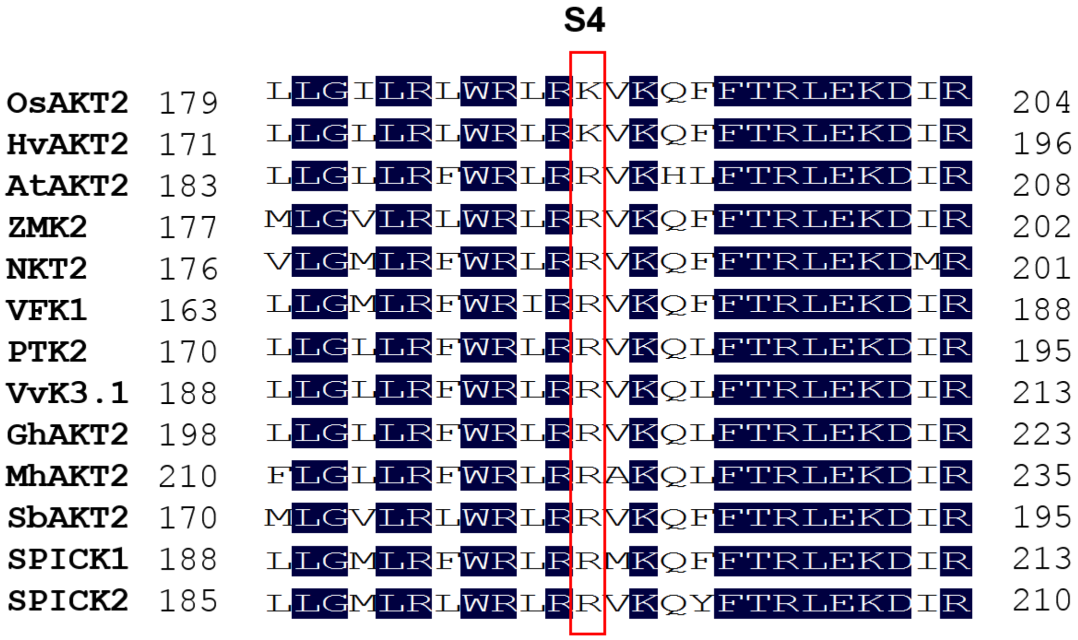

Supplemental Figure S4. S4 amino acid sequence comparison among known AKT2 channels.

Plant species and corresponding AKT2 channels analyzed were: *Oryza sativa*, OsAKT2 (JN989970); *Hordeum vulgare*, HvAKT2 (DQ465923); *Arabidopsis thaliana*, AtAKT2 (AAA97865); *Zea mays*, ZmK2.1 (AJ132686); *Nicotiana tabacum*, NKT2 (AB196789); *Vicia faba*, VFK1(Y10579); *Populus tremula*, PTK2 (AJ271447.1); *Vitis vinifera*, VvK3.1 (XM_002268888.3); *Gossypium hirsutum*, GhAKT2 (NM_001327179); *Malus hupehensis*, MhAKT2 (JN632525); *Sorghum bicolor*, SbAKT2 (XM_002441096); *Samanea saman pulvinus*, SPICK1 (AF099095) and SPICK2 (AF145272). The red frame highlighted the distinct residue we focused on in this study.

Supplemental Table S1. Primer sequences used in this study.

**Primer name**	**Primer sequence (5’- 3’)**
**Primers used for OsAKT2 clone**	
OsAKT2-F	GGCCCGGGATGAAGACCTCGAGCTTC
OsAKT2-R	TCCGCGGCCGCCTATGATCCAGACACCGAGTCCA
**Primers used for site-directed mutagenesis construction**	
OsAKT2-S4$^{(AtAKT2)}$-F1	cgtctcaggagagtcaagcatctcTTCACAAGGCTGGAGAAGGACA
OsAKT2-S4$^{(AtAKT2)}$-R1	ttgactctcctgagacgccagaaccggagtaaGCCGAGGAGGCTGAACGC
OsAKT2-S4$^{(AtAKT2)}$-F2	CAGGtactctTACTTCTGGATCCGCTGCGCCCG
OsAKT2-S4$^{(AtAKT2)}$-R2	CAGAAGTAagagtaCCTGATGTCCTTCTCCAGCCT
OsAKT2-Pore$^{(AtAKT2)}$-F1	CACCgcagcaatcTACTGGTCCATCACCACCATGA
OsAKT2-Pore$^{(AtAKT2)}$-R1	AGTAgattgctgcGGTGTAGCGGATCCACAGGC
OsAKT2-Pore$^{(AtAKT2)}$-F2	cacgcctcaaacaccatcGAGATGATCTTCAACATCTTCTACATGC
OsAKT2-Pore$^{(AtAKT2)}$-R2	atggtgtttgaggcgtgcaaGTCGCCGTAGCCGACGGT
OsAKT2-K193I-F1	cgagaattcacgcgtggtaccATGAAGACCTCGAGCTTCGAGA
OsAKT2-K193I-R1	tccagccttgTGAAGAACTGTATGACCTTCCTGAGA
OsAKT2-K193I-F1	cagttcttcaCAAGGCTGGAGAAGGACATCA
OsAKT2-K193I-R2	tcatgtctgctcgaagcggccgcCTATGATCCAGACACCGAGTCCA
OsAKT2-R190K-F	GCGTCTCaagAAGGTCAAGCAGTTCTTCACAAGG
OsAKT2-R190K-R	TGACCTTcttGAGACGCCATAGCCGGAGGATG
OsAKT2-K191R-F	AGGcgaGTCAAGCAGTTCTTCACAAGGCTGGA
OsAKT2-K191R-R	AACTGCTTGACtcgCCTGAGACGCCATAGCCGG
OsAKT2-Q194H-F	GGAAGGTCAAGcatTTCTTCACAAGGCTGGAGAAGG
OsAKT2-Q194H-R	GAAatgCTTGACCTTCCTGAGACGCCATAGCC
OsAKT2-L186F-F	ttcTGGCGTCTCAGGAAGGTCAAGCAGTTCTT
OsAKT2-L186F-R	TTCCTGAGACGCCAgaaCCGGAGGATGCCGAGGAG

OsAKT2-R190K-K191R-F TCTCaagcgaGTCAAGCAGTTCTTCACAAGGCT

OsAKT2-R190K-K191R-R TGCTTGACtcgcttGAGACGCCATAGCCGGAGG

OsAKT2-ANK$^{(OsAKT1)}$-F1 cgagaattcacgcgtggtaccATGAAGACCTCGAGCTTCGAA

OsAKT2-ANK$^{(OsAKT1)}$-R1 aattggcaaatcGCCGTGGTCGTACTCGCC

OsAKT2-ANK$^{(OsAKT1)}$-F2 accacggcGATTTGCCAATTACACTCTGTTTTG

OsAKT2-ANK$^{(OsAKT1)}$-R2 agtcgacgacgatggtACTCGCCGTCGCCGCCTT

OsAKT2-ANK$^{(OsAKT1)}$-F3 gagtACCATCGTCGTCGACTCGC

OsAKT2-ANK$^{(OsAKT1)}$-R3 tcatgtctgctcgaagcggccgcCTATGATCCAGACACCGAGTCCA

OsAKT1-ANK$^{(OsAKT2)}$-F1 cgagaattcacgcgtggtaccATGGCGAGGTGGGGCGCC

OsAKT1-ANK$^{(OsAKT2)}$-R1 cggtatgttCAGATTTCCCCGAGCTAGCA

OsAKT1-ANK$^{(OsAKT2)}$-F2 ggggaaatctgAACATACCGTGCAATCTGCTGA

OsAKT1-ANK$^{(OsAKT2)}$-R2 tgggacgtgatggtggccGACCGGGTGCGCGAGCTC

OsAKT1-ANK$^{(OsAKT2)}$-F3 tcGGCCACCATCACGTCCCA

OsAKT1-ANK$^{(OsAKT2)}$-R3 tcatgtctgctcgaagcggccgcGCTCTTGCCTTTCATCTTCTCTG

AtAKT2-F cgagaattcacgcgtggtaccATGGACCTCAAGTATTCAGCATCTC

AtAKT2-R tcatgtctgctcgaagcggccgcCTAAATTATCTTGTTTACGACAAAGTAGAGTT

AtAKT2-S4$^{(OsAKT2)}$-F1 atggcgacttcgaaaggttaaacagttcTTCACTAGGCTCGAGAAGGACAT

AtAKT2-S4$^{(OsAKT2)}$-R1 cctttcgaagtcgccatagtctaaggatTCCCAAGAGATTACAAGTGATATTTAACG

AtAKT2-S4$^{(OsAKT2)}$-F2 AGAttcaacTATTTCTGGATTCGCTGCTTTCGA

AtAKT2-S4$^{(OsAKT2)}$-R2 CCAGAAATAgttgaaTCTTATGTCCTTCTCGAGCCTAGTG

AtAKT2-R194K-F GGCGACTTaagAGAGTTAAACACCTCTTCACTAGGCTC

AtAKT2-R194K-R AACTCTcttAAGTCGCCAAAATCTAAGTAATCCC

AtAKT2-R195K-F GCGACTTCGAaagGTTAAACACCTCTTCACTAGGCTCG

AtAKT2-R195K-R TAACcttTCGAAGTCGCCAAAATCTAAGTAAT

AtAKT2-R194K-R195K-F GGCGACTTaagaagGTTAAACACCTCTTCACTAGGCTCG

AtAKT2-R194K-R195K-R TAACcttcttAAGTCGCCAAAATCTAAGTAATCCC

Primers used for qPCR

OsAKT2-qF AGCCGTCATCCGGGAGGTCGGC

OsAKT2-qR TCAATCTCCGCTCCTTCGTCGTT

OsActin-qF TGGTCGTACCACAGGTATTGTGTT

OsActin-qR AAGGTCGAGACGAAGGATAGCAT

OsCBL1-qF CGATGGGCTCATCAATAAGG

OsCBL1-qR CCCCCTTTTCTTCACATCAA

OsCIPK23-qF GCCTCCAAGGTTTGAGACAG

OsCIPK23-qR GCCAAGATTGAGACCCTGAG

Primers used for promoter clone

OsAKT2-promoter-F gagctcggtacccggggatccCAGCTTGCCCAGGTCGAGG

OsAKT2-promoter-R ttaccctcagatctaccatggTGCTACCAGAAGACGTTGCACA

Primers used for eGFP fluorescence observation

OsAKT2-eGFP-F1 cgagaattcacgcgtggtaccATGAAGACCTCGAGCTTCGAGA

OsAKT2-eGFP-R1 tgctcaccatTGATCCAGACACCGAGTCCATC

OsAKT2-eGFP-F2 gtctggatcaATGGTGAGCAAGGGCGAGG

OsAKT2-eGFP-R2 tcatgtctgctcgaagcggccgcTCACTTGTACAGCTCGTCCATGC
